# Fertility Concerns and Outcomes Among Adolescent and Young Adult Males With Melanoma Receiving Immunotherapy: A Mixed‐Methods Study

**DOI:** 10.1002/cam4.71437

**Published:** 2025-11-29

**Authors:** Haadi Ali, Ellen Wold, Tarek Haykal, Peter Dawood, Victoria Wytiaz

**Affiliations:** ^1^ Division of Medical Oncology, Department of Medicine University of Michigan Ann Arbor Michigan USA; ^2^ Rogel Cancer Center University of Michigan Ann Arbor Michigan USA; ^3^ Department of Medicine University of Michigan Ann Arbor Michigan USA; ^4^ University of Michigan Ann Arbor Michigan USA

## Abstract

**Introduction:**

Immune checkpoint inhibitors (ICIs) are often the initial treatment choice for melanoma, the third most common cancer in the adolescent and young adult (AYA) male population. Although ICIs have improved survival rates, their effects on male fertility and the adequacy of related counseling remain unclear. This study aimed to both characterize the current state of fertility discussions in male AYA patients with melanoma receiving first‐line ICIs and to explore their experiences and concerns regarding reproductive health.

**Methods:**

We conducted a mixed‐methods study of 38 male patients aged 18–39 with metastatic or locally advanced melanoma treated with first‐line PD‐1/PD‐L1/CTLA‐4 therapies at a single academic center between 2013 and 2024. Quantitative medical record review assessed fertility counseling and preservation actions. Semi‐structured interviews were conducted with ten living patients from this cohort to explore knowledge, concerns, and experiences surrounding fertility and reproductive health. Thematic analysis identified key patient perspectives.

**Results:**

Among 38 patients (median age 32), 71% had stage IV disease and received combination immunotherapy. 74% of patients experienced at least one immune‐related adverse event (irAE). Fertility counseling was documented for 27 patients, with eight referred for fertility preservation. Of the ten interviewees, nine recalled discussing fertility, while only three pursued preservation. Qualitative themes included challenges accepting uncertainty about family planning, pressure to accelerate planning, feelings of forced maturity, difficulty processing complex information, and concerns about the reliability of information from family, providers, and social media.

**Conclusion:**

Although most young males with advanced melanoma treated with first‐line ICIs had documented fertility discussions, few elected fertility preservation. Patient experiences highlight the need for collaboration among oncologists, fertility specialists, and supportive professionals to develop tailored counseling and educational materials for male AYA patients with advanced melanoma.

## 1 Background

Adolescent and young adults (AYAs), defined as individuals aged 15–39 years, are experiencing an increasing incidence of melanoma, making survivorship and long‐term quality of life key issues for this demographic [1, 2, 3, 4, 5, 6]. In terms of outcomes, immune checkpoint inhibitors (ICIs) have transformed melanoma treatment, improving disease control and survival rates. Recent studies have shown that ICIs have increased five‐year survival rates in patients with metastatic melanoma to nearly 50%. ICIs are also increasingly being used in neoadjuvant and adjuvant settings in patients with earlier, potentially curable disease [7]. ICIs are therefore often the first‐line systemic agents for advanced melanoma, meaning that many AYA males with advanced melanoma receive ICIs during a phase of life when fertility and future family planning are particularly important. Concerns related to fertility, including fears of infertility, the negative impact on romantic relationships, and the ability to successfully parent and raise children often arise at the time of any cancer diagnosis during the AYA period and this can persist into survivorship [2, 8]. Oncology providers recognize the risk of infertility in the AYA cancer population, and this possibility is often included in the consent process for cytotoxic chemotherapy and ideally further addressed by a reproductive specialist [9].

While validated measures have been developed to better assess cancer‐related reproductive concerns in the AYA cancer population in general, there is a paucity of data regarding the specific impact of ICIs on male fertility [10]. Current evidence is sparse and at times conflicting, with reports of immune‐related adverse events (irAEs) including hypogonadism, orchitis, azoospermia, erectile dysfunction, diminished endocrine function, and impaired semen parameters [11, 12, 13, 14, 15, 16]. The lack of evidence compounds the inherent uncertainty that exists with a cancer diagnosis at a young age and makes it challenging for clinicians to provide appropriate counseling prior to ICI initiation. This uncertainty also has a significant impact on timely decision‐making regarding sperm banking and fertility preservation, which often carry significant out‐of‐pocket costs and must be pursued within a limited window before treatment initiation.

This study addresses these critical gaps by integrating quantitative and qualitative methods to assess real‐world fertility counseling and preservation practices, and to explore reproductive health knowledge, concerns, and experiences of young male patients with advanced melanoma treated with ICIs at a single tertiary care center.

## 2 Methods

### 2.1 Study Design

This was a mixed‐methods study integrating retrospective quantitative analysis with qualitative interviews to comprehensively examine fertility counseling and reproductive concerns among AYA males with advanced melanoma treated with ICIs in the first‐line setting.

### 2.2 Quantitative

We (HA, EW, TH, VW) reviewed the electronic health record (EHR) of all male patients ages 18–39 with a diagnosis of melanoma who received first‐line therapy with an immunotherapy agent between January 1, 2013 and December 31, 2024. Patients were excluded from the study if they received another systemic melanoma‐directed therapy prior to immunotherapy, had a diagnosis other than melanoma, or were taking medications that could independently impact fertility.

Data collected included: patient‐related factors, melanoma‐related data (diagnosis, stage), treatment‐related information (immunotherapy regimen, indication, side effects, prior surgery or location therapy), and fertility‐related factors (children prior to or after starting immunotherapy, formal fertility consultation, fertility preservation or fertility preservation discussions).

Descriptive statistics were utilized to summarize melanoma characteristics, patient demographics, immunotherapy regimens, treatment toxicities, and fertility‐related outcomes. Categorical variables were reported as counts and percentages. Continuous variables, including age at diagnosis and ICI initiation, were reported via medians and ranges. All statistical analyses were performed using Microsoft Excel 365.

### 2.3 Qualitative

Following quantitative data collection, we conducted semi‐structured interviews with a random cohort of 10 living patients from the identified population described above. Randomization was performed utilizing the data analysis tools of Microsoft Excel. Interview content was comprised of three major sections that addressed participant knowledge of infertility risk, concerns and needs related to fertility, and the availability or utilization of fertility preservation services. Examples of specific probes used by the authors conducting the interviews (VW, HA, EW) included describing emotional reactions to learning about infertility risk, satisfaction with the discussion of these risks, and reasons for pursuing or not pursuing fertility preservation. Following the conclusion of each interview, participants were provided with a $20 gift card. The local Institutional Review Board approved this study (HUM#00269570).

Interviews were professionally transcribed by a HIPAA‐compliant transcription service. The authors (V.W., H.A., E.W.) independently coded the 10 transcripts using a deductive coding schema derived from the empirical literature addressing information needs [17, 18], knowledge about treatment effects [18, 19, 20], and reproductive concerns after cancer [21, 22] (see Table 1). Coders met to discuss and resolve discrepancies. Final codes were compiled into a master document, and this was also reviewed for emergent, inductive themes.

**TABLE 1:** Deductive coding schema.

Code	Definitions
Reproductive concerns	Acceptance of potential infertility
	Disclosing fertility status with partners
	Costs of fertility preservation
Information needs	Information from medical providers
	Information from other patients
	Internet/social media resources
Knowledge of effects	Treatment factors affecting fertility
	Reproductive technology and fertility preservation
	Alternative parenting options (adoption, child‐free living, surrogacy)

## 3 Results

### 3.1 Quantitative

Patient demographics and clinical characteristics are summarized in Table 2. The study cohort included 38 male patients, of whom 29 (76%) were alive at the time of data collection, with a median age of 32 years at diagnosis (range, 17–39) and 34 years at ICI initiation (range, 19–39). Most patients had cutaneous melanoma (55%), with additional subtypes including nodular (13%), unknown primary (21%), mucosal (5%), and other variants (5%). BRAF V600E mutations were present in 66% of patients, 32% were BRAF wildtype, and one patient's BRAF status was unknown. At the time of ICI initiation, the majority of patients had advanced disease, with 71% at stage IV and 29% at stage III.

**TABLE 2:** Clinical, treatment, toxicity, and fertility characteristics of young male melanoma patients treated with immune checkpoint inhibitors (*n* = 38).

All patients (*n* = 38)	
*n* (%)	
Median age (range)	
At diagnosis	32 (17–39)
At ICI start	34 (19–39)
Sex	
Male	38 (100%)
Survival status	
Alive	29 (76%)
Deceased	9 (24%)
Melanoma characteristics	
*Subtype*	
Cutaneous	21 (55%)
Nodular	5 (13%)
Mucosal	2 (5%)
Unknown primary	8 (21%)
Other	2 (5%)
*BRAF mutation status*	
Wildtype	12 (32%)
V600E	25 (66%)
Unknown	1 (3%)
*Stage at ICI initiation*	
1	0 (0%)
2	0 (0%)
3	11 (29%)
4	27 (71%)
ICI therapy	
*Regiments used*	
Ipilimumab/Nivolumab	27 (71%)
Nivolumab/Relatlimab	7 (18%)
Ipilimumab	1 (3%)
Nivolumab	22 (58%)
Pembrolizumab	12 (32%)
*Purpose of ICI therapy*	
Neoadjuvant	5 (13%)
Adjuvant	1 (3%)
Metastatic disease	32 (84%)
*Subsequent lines of immunotherapy*	
0	12 (32%)
1	22 (58%)
2 or more	4 (11%)
ICI toxicities	
*Total number of irAE*	
0	10 (26%)
1	19 (50%)
2	4 (11%)
3	4 (11%)
4 or more	1 (3%)
*irAE grading*	
Grade 1–2	14 (37%)
Grade 3–4	12 (32%)
*Endocrine irAE*	
Thyroiditis	6 (16%)
Hypophisitis	2 (5%)
Children	
*Number of children pre‐ICI treatment*	
0	20 (53%)
1	6 (16%)
2	7 (18%)
3 or more	5 (13%)
*Children conceived post‐ICI*	
Yes	1 (3%)
No	29 (76%)
Unknown	8 (21%)
Fertility	
Fertility preservation discussed	29 (76%)
Fertility preservation referral placed	8 (21%)
Fertility preservation performed	4 (11%)

Combination ipilimumab/nivolumab was the most frequently used regimen (71%), with additional regimens including nivolumab/relatlimab (18%), ipilimumab monotherapy (3%), nivolumab monotherapy (58%), and pembrolizumab (32%). Because some patients received multiple ICI regimens sequentially, these categories are not mutually exclusive. Most patients (84%) received immunotherapy for metastatic disease, while 13% were treated in the neoadjuvant setting and 3% in the adjuvant setting. Regarding additional treatment, 58% of patients received one subsequent line of immunotherapy and 11% received two or more.

irAEs occurred in 74% of patients, with grade 3–4 irAEs observed in 32%. Endocrine irAEs included thyroiditis in 16% and hypophysitis in 5% of the cohort. More than half of patients (53%) were childless before ICI initiation, while 16% had one child, 18% had two, and 13% had three or more children.

Fertility preservation was discussed with 76% of patients, referrals for preservation were placed in 21%, and preservation procedures were performed in 11%. Our dataset did not capture patient‐reported reasons for declining referral or preservation, which limits the interpretation of these findings and highlights the need for further research to understand patient decision‐making and barriers to fertility preservation in this population.

### 3.2 Qualitative

Of the 38 patients in the cohort, ten living patients were randomly selected for semi‐structured interviews. Among these interviewees, nine patients recalled discussing fertility with their oncology team while three patients completed fertility preservation. Six patients disclosed that they had children prior to treatment, and three had healthy children post‐treatment. No patients utilized banked sperm from their fertility preservation process. Analysis of interview transcripts revealed three themes: (1) challenges in accepting the uncertainty of future family planning (2) feelings of accelerated timelines and maturity, and (3) concerns about reliable ingoing and outgoing information. Representative quotations are shown in Table 3.

**TABLE 3:** Representative quotation of emergent themes.

Challenges in accepting the uncertainty of future family planning
*“We [patient and wife] knew the plan was always to get married and have kids. I mean, not everyone, but most people, I feel like they want a little mini me, like a little kid. I know people adopt, but I just feel like it's not the same.”*
*“I wasn't aware of any risks as far as fertility but that's probably because we [patient and wife] weren't thinking of it at the time. Because, yeah, I don't even remember talking too much about that kind of risk. I always have the mindset of like, this is just going to work. Everything's going to be fine.”*
*“There was always that kind of concern in the background as far as like, I hope everything [conceiving naturally] goes okay. But really, it was like, is this gonna work? Or are we gonna have to do the sperm bank kind of thing? So we weren't sure.”*
*“And if I can't have kids, I mean that's*… *There goes my whole relationship with [my wife]. How does that look if she stays with me for 10 years and we don't have kids. I mean, it's terrible. It was the biggest fear I had ‘cause I'm not only gonna have cancer, but I also potentially put a huge strain on the most important relationship in my life.”*
“*I had a conversation with [the oncologist] and they said, we don't really know what the long‐term effects or if it has any effect on fertility or what have you. So I went to the fertility clinic and banked some specimens and that was successful. And so I had that kind of in reserve, so to speak, in the event that I couldn't have kids.”*
Feelings of accelerated timelines and maturity
*“My plan was for us [patient and girlfriend] to go through nursing school, propose, get married. Super*… *So, get married after nursing school, then have kids. And then when this diagnosis came…I said [to my girlfriend], do you wanna have a baby before I start treatment? And she's like yeah.”*
*“And it was like I always had career goals, like five‐year goals was my thing since, I don't know, I've been 30. And it's always worked out pretty well, I put a lot into my career*. *And I would say after [immunotherapy], it definitely changes your mindset. It's not that I don't care about work, but I think a lot of your goals shift toward your personal life. You're much more needed at home than you are at work.”*
*“I don't know if I had a 5‐to‐10‐year plan before the diagnosis. I mean, I was literally in the front half of college. I think I've always known I wanted to have kids, but I would tell you that even now, I don't think that's anytime soon, probably mid‐30s maybe. But yeah, that wasn't even at all in my near future.”*
*“Yeah, having kids was kind of on the radar and I had just gotten a new job not too long ago. I'd moved to a new area, and we were trying to get settled. We were just trying to figure out, when was the right time and if and when and how and all that kind of stuff. So, had a conversation and said, hey, well, let's try now.”*
*“Definitely not [thinking of having kids] at that time. I mean, I think I've always known I wanted to have kids, but I would tell you that even now, I don't think that's anytime soon, probably mid‐30s maybe. But yeah, like it wasn't a thought, not in the near future at all.”*
Concerns about reliable ingoing and outgoing information
*“I mean, it was an overload of information in general during that time in my life because there was a lot going on. Because it is like a total mind bender to have somebody tell you that you have cancer and there is a risk if infertility. And the worst thing you can do is go online because it's like anything's wrong with you and you go online, it's a death sentence.”*
*“I thought it [treatment] was chemotherapy. I didn't know that melanoma was immunotherapy, not chemo. What do you do? You Google chemo. Well, if you have chemo, you don't know how that's gonna affect your fertility, all these things.”*
*“When I was diagnosed, I didn't have a really super good understanding of immunotherapy* versus *chemo* versus *other treatments. It's very new. At that time, it had been around really less than 10 years, so there wasn't a lot of data. From there, it's like, “Am I going to die? Am I not going to die?” I don't know. I'm just going to do what I'm told. We'll see what happens.”*
*“I feel like [information] is more legit when it's written in a research paper* vs. *somebody on YouTube telling me something. So that's what I tried to stick to, but it's very hard when, you know, the AI would spit out, you know, death rates and like their survival rates. And those were horrible things to see. But I feel like everybody's going to look at that.”*
*“I would have sought a second opinion and then said okay, both of you agree and you're both qualified people who understand this, then I would go for it. I'm an engineer originally. That's probably a little bit of what this sort of thought process and training comes from in terms of, I understand where I'm an expert and where I'm not an expert. I also understand that sort of the scientific methodology process and, okay, let's go get some expert advice.”*

#### 3.2.1 Theme 1: Challenges in Accepting the Uncertainty of Future Family Planning

Many patients experienced emotional distress and uncertainty about future family planning. One explained, “We [patient and wife] knew the plan was always to get married and have kids. I mean, not everyone, but most people, I feel like they want a little mini me, like a little kid. I know people adopt, but I just feel like it's not the same.” Others worried about how infertility may impact relationships, as demonstrated by one interviewee, “There goes my whole relationship with [my wife]…It was the biggest fear I had cause I'm not only gonna have cancer, but I also potentially put a huge strain on the most important relationship in my life.”

#### 3.2.2 Theme 2: Feelings of Accelerated Timelines and Maturity

Participants recounted that their diagnosis and treatment significantly altered their life plans and priorities. For example, one patient stated, “My plan was for us [patient and girlfriend] to go through nursing school, propose, get married. And then when this diagnosis came I said [to my girlfriend], do you wanna have a baby before I start treatment? And she's like yeah.” For others, the melanoma diagnosis shifted their priorities from career to family. As depicted by another patient, “… after [immunotherapy], it definitely changes your mindset. It's not that I don't care about work, but I think a lot of your goals shift toward your personal life. You're much more needed at home than you are at work.”

#### 3.2.3 Theme 3: Concerns About Reliable Ingoing and Outgoing Information

Difficulty navigating the amount of available information was distressing for patients. One shared, “It was an overload of information in general during that time in my life…And the worst thing you can do is go online because it's like anything's wrong with you and you go online, it's a death sentence.” Patients also experienced confusion about their specific treatment regimens. One explained, “I thought it [treatment] was chemotherapy. I didn't know that melanoma was immunotherapy, not chemo…” Others expressed difficulty determining the legitimacy of sources, particularly online resources. As demonstrated by one patient, “*I feel like [information] is more legit when it's written in a research paper* vs. *somebody on YouTube…but it's very hard when, you know, the AI would spit out, you know, death rates and like their survival rates. And those were horrible things to see*.”

## 4 Discussion

Our mixed‐methods study examined fertility‐related outcomes in AYA males who received ICI therapy by integrating retrospective chart review with patient interviews to evaluate practice patterns and patient experiences. Our findings highlight gaps in the clinical space as young males consider undergoing fertility preservation and this is further supported by patient interview content emphasizing the emotional and informational complexities that patients experience with fertility‐related decision‐making.

Most patients in our single‐center retrospective cohort study were diagnosed in early adulthood (median age, 32 years), and 71% of patients presented with stage IV melanoma at the time of ICI initiation. Dual ipilimumab and nivolumab, which is the current standard of care, were the most frequently used regimens for advanced‐stage melanoma. In our cohort, irAEs effects were common, occurring in 74% of patients. 32% of patients experienced endocrine‐related irAEs such as thyroiditis (16%) and hypophysitis (5%). These off‐target effects are known to contribute to infertility through disruption of the hypothalamic–pituitary–gonadal axis.

Despite these complications, fertility preservation was underutilized. While 76% of patients, upon record review, had documented confirmation of fertility discussions, only 21% were referred to fertility preservation specialists, and only 11% completed preservation procedures, specifically sperm cryopreservation. These rates differ from those observed in studies evaluating fertility counseling and preservation in young male patients with any cancer subtype or treatment modality posing a fertility risk. One such study showed that 43% of all males ages 18–40 with cancer received fertility consultation [23] while another study revealed that 28% of all adolescent male patients with cancer pursued sperm cryopreservation [24]. The lower utilization of both consultation and preservation services in our study may be due in part to the uncertainty of long‐term fertility‐related complications with immunotherapy when compared to those that are well established with chemotherapy.

Furthermore, this discrepancy also suggests systematic barriers between counseling and intervention in this treatment space that serves a unique population. Potential strategies to improve these care gaps include collaboration between oncologists, fertility specialists, and clinical support staff to tailor specific educational materials and provide individualized counseling that serves the needs of the male AYA population. Another barrier is treatment urgency as noted in our qualitative work which can be addressed by integrating sperm collection and fertility preservation soon after diagnosis. Significant emotional burden is likely present as highlighted by our qualitative findings emphasizing the role of clinical psychology early on following melanoma diagnosis.

Qualitative interviews with ten AYA male melanoma survivors further contextualized the disparity between fertility counseling and intervention. While nine patients underwent fertility discussions, only three patients pursued preservation. Interestingly, none underwent sperm banking. Despite receiving immunotherapy, three patients fathered healthy children following immunotherapy, highlighting a potential recovery in spermatogenesis following ICI treatment. Even in other malignancies with higher preservation rates, utilization rarely exceeds 30% [25, 26]. Multiple factors contribute to sperm bank underutilization. In our cohort, one patient explained that he and his partner decided against having children before his cancer diagnosis, highlighting wanted childlessness. Notably, no participants reported infertility or unwanted childlessness. However, one shared that cancer‐related stress led to him and his partner having fewer children than originally planned. Unlike systemic chemotherapy, endocrine‐related toxicities contributing to infertility with ICIs are not highlighted [27, 28] as supported by qualitative interviews. Moreover, educating practicing oncologists on 2025 American Society of Clinical Oncology (ASCO) guidelines about fertility management before melanoma treatment will standardize provider and patient perception of future fertility planning. Collaborative patient‐clinician education coupled with integrating reproductive specialists and clinical psychology is a crucial component of the care plan for AYA males undergoing melanoma treatment as emphasized in the pediatric setting, in which prompt multidisciplinary fertility counseling integrating family and significant others was shown to improve patient outcomes and overall satisfaction following treatment [25, 26, 29].

Our qualitative work also emphasized themes of uncertainty regarding future family planning, with a recurring emphasis on accelerated timelines for family decision‐making. There remains a significant gap in understanding how the effects of immunotherapy on fertility differ from those of systemic chemotherapy. While our center routinely offers semen preservation before ICI initiation, this is not standard practice. Current guidelines primarily focus on fertility preservation in those receiving gonadotoxic chemotherapy or radiotherapy [27, 28]. Notably, in our cohort, none of the three patients who underwent sperm banking utilized their samples—two conceived naturally, and the third had not yet considered childbearing. These findings raise questions about the utility of sperm banking in this unique population. While a recent prospective study found that most men treated with ICIs experienced only minimal and transient changes in sperm function, there remains an unpredictable 4% risk of developing severe, potentially irreversible testicular dysfunction due to autoimmune orchitis [30]. In our cohort, the patients who were able to conceive did not experience irAE, including autoimmune orchitis. This unpredictability highlights the importance of individualized survivorship planning and the need for further research to clarify the long‐term effects ICIs have on fertility to develop evidence‐based recommendations for AYA males with melanoma.

This study has several limitations. First, our qualitative analysis is based on interviews with only ten patients and may not reflect the full range of experiences within the AYA melanoma population. The retrospective design and reliance on existing documentation could also limit the accuracy and completeness of fertility counseling and outcome data. We were also unable to assess long‐term fertility outcomes following ICI therapy due to limited follow‐up duration as highlighted by 3 patients conceiving children following ICI therapy. These findings further emphasize the gap between fertility preservation counseling and utilization. Future studies are needed to better understand patient decision‐making, barriers to fertility preservation and long‐term reproductive outcomes following ICI therapy.

## 5 Conclusion

Our single cohort retrospective chart review based in a tertiary center demonstrates that while fertility discussions are occurring, preservation rates remain suboptimal for AYA males undergoing immunotherapy for advanced‐stage melanoma. Our qualitative analysis highlights the urgent need for prompt and emotionally targeted fertility counseling soon after diagnosis. Integration of reproductive specialists into oncology care and long‐term follow‐up may contribute to improved adherence to sperm banking and overall quality of life measures. To address these gaps in care, targeted interventions should be studied to enhance sperm banking rates and follow up on utilization following the completion of melanoma treatment. Other areas that should be explored include long‐term fertility outcomes following immunotherapy.

## Author Contributions

**Haadi Ali:** writing – review and editing, resources, investigation, writing – original draft, methodology, conceptualization, formal analysis. **Ellen Wold:** investigation, writing – original draft, methodology, writing – review and editing, formal analysis, resources, data curation. **Tarek Haykal:** conceptualization, investigation, methodology, validation, writing – review and editing, formal analysis, data curation, supervision. **Peter Dawood:** writing – original draft, resources, writing – review and editing. **Victoria Wytiaz:** conceptualization, investigation, funding acquisition, writing – original draft, methodology, writing – review and editing, formal analysis, supervision, resources.

## Funding

The authors have nothing to report.

## Ethics Statement

This study was approved by the Institutional Review Board of the University of Michigan Medical School (IRB Protocol Number: HUM#00269570).

## Consent

Consent was obtained from all interview participants.

## Conflicts of Interest

The authors declare no conflicts of interest.

## Data Availability

The data that support the findings of this study are not publicly available due to privacy or ethical restrictions.
